# From mouse to human

**DOI:** 10.7554/eLife.94382

**Published:** 2023-12-07

**Authors:** Arya Mani

**Affiliations:** 1 Department of Internal Medicine and Genetics, Yale University School of Medicine New Haven United States

**Keywords:** atherosclerosis, coronary artery disease, gene regulatory networks, multiomics, cross-species comparison, genetics, Human, Mouse

## Abstract

A deep analysis of multiple genomic datasets reveals which genetic pathways associated with atherosclerosis and coronary artery disease are shared between mice and humans.

**Related research article** Kurt Z, Cheng J, McQuillen CN, Saleem Z, Hsu N, Jiang N, Barrere-Cain R, Pan C, Franzen O, Koplev S, Wang S, Bjorkegren J, Lusis AJ, Blencowe M, Yang X. 2023. Shared and distinct pathways and networks genetically linked to coronary artery disease between human and mouse. *eLife* **12**:RP88266. doi: 10.7554/eLife.88266.

Over time, various substances that travel through blood – such as cholesterol, inflammatory cells, and cellular debris – can accumulate in the walls of arteries, resulting in their narrowing. This build-up of materials, known as atherosclerosis, can also occur in the blood vessels that supply nutrients and oxygen to the heart, leading to coronary artery disease. Understanding what causes atherosclerosis is crucial for developing effective preventive and therapeutic strategies for coronary artery disease.

Genome-wide association studies – which compare common DNA variations in populations with and without a specific trait or disease – have identified numerous genetic variants linked with an increased risk of atherosclerosis (Khera and Kathiresan, 2017; Tcheandjieu et al., 2022). These variants are either causal or associated with various aspects of atherosclerosis, such as lipid metabolism, inflammation, and endothelial function. Despite significant advances in genetic research, it remains unclear which of these variants drive the condition, and in which genes and/or tissues these variants exert their effects. How other factors that are known to influence atherosclerosis, such as environment, sex and lifestyle, impact gene expression also cannot be inferred from these types of investigations.

To overcome these limitations, researchers use animal models that have been manipulated to develop a certain disease. Mice are the most commonly studied species, and have been used to observe how altering specific genes and controlling various environmental factors affect the way atherosclerosis and coronary artery disease develop. However, mice and humans differ significantly in terms of their physiology and genetics. For instance, their lipid metabolism and immune responses vary, and certain genes implicated in mice might not have direct equivalent functions or effects in humans, making it difficult to translate finding from studies in mice to clinical applications. Now, in eLife, Montgomery Blencowe and Xia Yang from the University of California, Los Angeles (UCLA) and colleagues – including Zeyneb Kurt and Jenny Cheng as joint first authors – report how the genetic pathways and mechanisms associated with atherosclerosis and coronary artery disease compare between these two species (Kurt et al., 2023).

Kurt et al. meticulously analyzed various sources of data, including mouse genomic data from the Hybrid Mouse Diversity Panel, and human genomic data from the CARDIoGRAMplusC4D consortium, GTEx database, and STARNET. In addition to results from genome-wide association studies (GWAS), these datasets include information on which genes are active and which variants alter the expression level of these genes (known as expression quantitative trait loci, or eQTL for short) in specific tissues of interest: the liver and vasculature tissues of humans, and the aorta (which is part of the vasculature) and liver tissues of mice.

First, the team (who are based at institutes in the United States, United Kingdom and Sweden) used the GWAS, gene expression, and eQTL data from mice and humans to determine which genes have similar expression profiles and are therefore likely to be connected, and which genes have a major role in the two conditions. Using these co-expression gene networks, together with another tool known as gene set enrichment analysis, they were able to identify the signaling pathways associated with coronary artery disease and atherosclerosis in humans and mice. Remarkably, this revealed a significant overlap in the pathways linked to coronary artery disease and atherosclerosis, with approximately 75% and 80% of identified pathways being associated with both diseases in the vasculature and liver tissue, respectively. These shared pathways encompass well-known processes, such as lipid metabolism, and introduce novel aspects like the mechanism that breaks down branched chain amino acids.

The analysis also uncovered pathways that were specific to each species, such as the insulin signaling pathway in the aorta of mice, and interferon signaling in the human liver. Kurt et al. then used a probabilistic model known as the Bayesian Network to pinpoint which genes were predominantly driving these species-specific pathways, and identified the subnetwork of genes immediately downstream or neighboring these drivers. The genes that drive the mouse-specific pathways were validated using single-cell RNA sequencing data, which revealed that the subnetwork of genes changed expression in the aortas and livers of mice with coronary artery disease and/or atherosclerosis.

Further analysis revealed that some of these previously unknown key driver genes were also hits in a recent GWAS of coronary artery disease, suggesting they have a crucial role in the disease. This included a key driver of coronary artery disease in both humans and mice, the *ARNTL* gene (also known as *BMAL1*) which is a transcriptional activator that forms a core component of the circadian clock and negatively regulates adipogenesis (Guo et al., 2012).

Interestingly, a common variant in the *ARNTL* gene has been associated with coronary artery disease and other factors linked to this condition and atherosclerosis, such as body mass index, diastolic blood pressure, triglyceride levels, and type 2 diabetes (van der Harst and Verweij, 2018; Pulit et al., 2019; Sakaue et al., 2021; de Vries et al., 2019, Vujkovic et al., 2020). Furthermore, values derived from the GTEx dataset suggest that the alternative variant reduces the expression of the gene in whole blood. Deletion of *ARTNL* in certain blood cells has also been shown to predispose mice to acute and chronic inflammation (Nguyen et al., 2013). Use of functional genomics, particularly in the context of sex differences, will likely establish the causality of *ARNTL* and other predicted key driver genes (Gunawardhana et al., 2023).

The findings of Kurt et al. are a pivotal contribution to our understanding of coronary artery disease and atherosclerosis in mice and humans. The integrative genomic study also creates avenues for further research, such as applying the same approach to larger GWAS datasets and incorporating variants that impact the splicing or quantity of protein produced into the analysis. Employing additional mouse models of atherosclerosis and coronary artery disease, and analyzing other relevant tissues, could also help identify additional cross-species similarities and differences. These future studies, together with the work by Kurt et al., will help researchers to determine how well findings in mice relate to human coronary artery disease and atherosclerosis, and whether these results could translate to clinical applications.
